# Reference intervals for hematology, serum biochemistry and blood gas parameters in Indian elephants (*Elephas maximus indicus*) under human care

**DOI:** 10.3389/fvets.2025.1602296

**Published:** 2025-07-14

**Authors:** Sanath Krishna Muliya, Uddalak Tathagato Bindhani, Lallianpuii Kawlni, Anjan Kumar Ramakrishna, Vaseem Mirza, Mujib Ur Rahman, Vinay Kumar, Ramesha Huchhaiah, Thammaiah Chekkera Kuttappa, Souritra Sharma, Kafil Hussain, Vishnupriya Kolipakam, Ramesh Kumar Pandey, Qamar Qureshi

**Affiliations:** ^1^Wildlife Institute of India, Dehradun, Uttarakhand, India; ^2^National Zoological Park & National Tiger Conservation Authority, Ministry of Environment, Forest and Climate Change, New Delhi, India; ^3^Forest Research Institute, Kaulagarh Road, Dehradun, Uttarakhand, India; ^4^Bangalore Veterinary College, Karnataka Veterinary, Animal and Fisheries Science University, Bangalore, India; ^5^Bandipur Tiger Reserve, Karnataka Forest Department, Mysore, Karnataka, India; ^6^Nagarahole Tiger Reserve, Karnataka Forest Department, Forest Campus, Mysore, Karnataka, India; ^7^Sakrebyle Elephant Camp, Karnataka Forest Department, Shivamogga, Karnataka, India; ^8^Sher-e-Kashmir University of Agricultural Sciences and Technology, Srinagar, India; ^9^Project Elephant, Ministry of Environment, Forest and Climate Change, New Delhi, India; ^10^Academy of Scientific and Innovative Research, Ghaziabad, India

**Keywords:** Asian elephants, blood gas, electrolytes, *Elephas maximus indicus*, hematology, physiological parameters, reference intervals, serum biochemistry

## Abstract

The Indian elephant (*Elephas maximus indicus*), classified as “Endangered” by the IUCN, faces significant population declines and habitat degradation, necessitating robust health assessment tools for both managed and free-ranging populations. This study aimed to establish comprehensive hematology, serum biochemistry and arterial blood gas reference intervals (RIs) for Indian elephants, addressing gaps in existing studies, and following the guidelines of the American Society for Veterinary Clinical Pathology (ASVCP). Samples were collected from a well-defined elephant population under human care in southern India and analyzed on the same day. Phlebotomy was carried out in unsedated elephants in lateral recumbency to avoid sedation-related effects on measurands. A total of 92 elephants were sampled. Hematology RIs were derived from EDTA whole blood using automated methods, while serum biochemistry RIs were generated using semi-automated analyzers; arterial blood gas analysis was also performed using a portable field analyzer. The established hematology RIs were as follows: Hb 8.62–16.78 g/dL, RBC 1.77–4.9 × 10^12^/L, PCV 21.73–49.25%, MCV 112.49–131.39 fL, MCH 39.3–62.39 pg./cell, MCHC 33.40–41 g/dL, platelet count 171.57–947.1 × 10^3^/μL, WBC 9912.5–29,475 cells/μL. Serum clinical chemistry RIs included: SGPT 4.01–20.34 U/L, ALP 124.89–556.68 U/L, TP 5.54–9.3 g/dL, albumin 2–2.91 g/dL, globulin 3.36–6.9 g/dL, GGT 2.38–23.18 U/L, CRT 0.65–2.06 mg/dL, BUN 4.12–24.32 mg/dL, and albumin/globulin ratio 0.3–0.8. Serum lipid profile RIs were: TC 26.96–69.39 mg/dL, triglycerides 12.55–52.47 mg/dL, HDL 9.02–39.38 mg/dL, LDL 4.63–49.07 mg/dL, and VLDL 2.52–10.5 mg/dL. Serum electrolyte RIs included: Ca 6.21–11.38 mg/dL, P 2.89–6.29 mg/dL, Na 133.94–174.77 mmol/L, K 1.83–7.81 mmol/L, and Cl 92.56–119.46 mmol/L. Arterial blood gas parameters were: pH 7.368–7.515, pCO₂ 28.5–47.9 mmHg, pO₂ 71.0–120.8 mmHg, cHCO₃^−^ 23–30.1 mmol/L, sO₂ 94.4–99.6%, and tCO₂ 22.8–29.5 mmol/L. These RIs provide critical baseline data for health monitoring, enabling the detection of subclinical infections and evaluation of physiological, nutritional, and ecological welfare. By enhancing our understanding of the species’ physiological norms, this study supports improved veterinary care and conservation strategies, ultimately contributing to the long-term survival and welfare of Indian elephants in the region.

## Introduction

The Indian elephant (*Elephas maximus indicus*) is one of the three recognized subspecies of the Asian elephant, native to mainland Asia. Its current habitat range spans across India, Nepal, China, Bangladesh, Bhutan, Myanmar, Thailand, Malaysia, Laos, Cambodia, and Vietnam (1). With an estimated population ranging from 26,000 to 28,000 individuals, approximately three-quarters of this subspecies are concentrated in mainland India (2, 3). The Indian elephant is classified as “Endangered” by the IUCN due to significant population decline, coupled with extensive habitat loss, fragmentation, and degradation (1). Recognizing its critical status, the subspecies is listed in Appendix I of the CITES and Schedule I of the Wildlife Protection Act (1972), affording it the highest levels of global and local protection, respectively, (4, 5). Indian elephants, in contrast to their African counterparts, also possess a long history of human utilization owing to their religious, cultural, and socioeconomic significance (6, 7). Presently, around 2,800 Indian elephants are housed in various governmental and private facilities in the country, making up roughly 20% of the global Asian elephant population held under human care (3, 8).

Wild animals in managed settings, particularly the species like elephants with unique physical, physiological and psychological needs, are prone to a host of health issues due to unnatural ex-situ conditions (9–11). Given the difficulty in diagnosing underlying health concerns in such megaherbivores, clinical pathology reference intervals play a crucial role in comprehensive health assessments, including detection of subclinical infections and evaluation of physiological, nutritional and ecological welfare (12–15). Reference intervals (RI) are particularly of relevance in managed elephant populations in India, as the availability of advanced diagnostic modalities is significantly limited due to either minimal infrastructure and remoteness of holding facilities, or the unaffordability of such diagnostic capabilities in facilities with fewer individuals.

Although numerous hematology and serum biochemistry ranges are available for Indian elephants, the analytical methods used in these studies are either undescribed or obsolete (16–20). Furthermore, these intervals are typically derived from limited population testing and do not consider the variations within subpopulations defined by biological factors such as age or gender and clinical factors such as physical, physiological and psychological health status. The available RIs for the subspecies, derived through modern analytics, are scarce and mostly from neighboring range countries having varying geography, climate, and ex-situ management conditions (21, 22).

The objective of this study was to establish reliable and robust RIs for hematology, serum biochemistry, and blood gas parameters in Indian elephants maintained under semi-captive human care in their native range, adhering to internationally recognized veterinary clinical pathology guidelines.

## Materials and methods

The study was carried out under the aegis of a larger project titled “Population Management of Species Involved in Human Wildlife Conflict” implemented following approvals from the Ministry of Environment, Forest and Climate Change, Government of India, as per the order no. 8-98/2016-WL, dated 30/01/2018. Permissions for elephant sampling were also specifically obtained from the Principal Chief Conservator of Forests and Chief Wildlife Warden, Karnataka through O.M No. PCCF (WL)/E (C1)/CR-03/2016-17 (07/06/2019), and No. PCCF (WL)/E (C1)/CR-03/2016-17 (09/12/2019), as per the existing rules under the Wildlife Protection Act, 1972, Government of India.

### Animals

To ensure a well-refined reference RI, the selection of individuals for the study was conducted in accordance with the guidelines provided by the American Society for Veterinary Clinical Pathology (ASVCP) (23). Elephants utilized for the study were all adults, healthy and housed under human care within the forest camps of the Karnataka Forest Department, situated in semi-natural conditions adjacent to protected areas or tiger reserves. While historically these camps housed elephants for logging and tourism purposes, their current activities are restricted to forest management tasks such as patrolling or serving as “Kumkis” to aid in the capture operations of other wild animals. These elephants are maintained under uniform husbandry conditions, with access to similar nutrition, physical activity, and natural habitat (24). They are both stall-fed and provided access to natural forage, spending at least 12 h per day in natural forests. Elephants with history of recent illness, injury, physical and phycological stress, as well as physiological variation such as musth, pregnancy and lactation were excluded from the study. Data for each individual elephant, such as name, estimated age, sex, overall condition, estimated weight, and the geographical location of the forest camp, were recorded using the Windows Excel program.

### Sampling and laboratory analysis

To mitigate significant fluctuations in environmental and climatic conditions, sampling of all the elephants was conducted within a six-month period, spanning from June 2021 to November 2021. Sample collection for each elephant was conducted according to a standardized protocol without the use of chemical immobilization, as follows: Sampling took place early in the morning, before the day’s first stall feeding. Elephants were made to lie down in lateral recumbency using oral cues from the caretaker/mahout. For hematology and biochemistry, blood was drawn through direct phlebotomy using a 10 mL syringe and an 18G needle from the auricular vein, following aseptic techniques. Whole blood was collected in Ethylenediaminetetraacetic acid (EDTA) and gel-based clot activator vacutainers. Serum samples were allowed to clot for at least 15 min, standing upright in a cooler box, before being centrifuged at 1000 g for 10 min in the field for serum separation. Whole blood and serum were then immediately dispatched to a designated laboratory under a cold chain and analyzed within 6 to 8 h of collection. To avoid analytical bias, hematology and serum biochemistry analysis were consistently performed in the same laboratory using the same automated analyzers (BC-2800 Vet, Mindray, Shenzhen 518,110, P.R.C for hematology and Mispa Nano Plus, Agappe Diagnostics Ltd., Mumbai - 400099, India for serum biochemistry) according to the manufacturer’s instructions. Though these analyzers and settings have not been validated for elephant blood, internal quality control was ensured by performing manufacturer-supplied quality control protocols before analyzing each batch. For blood gas analysis, arterial blood was collected by aseptic techniques using a 1 mL syringe and 21 G needle from the auricular artery. Blood gas analysis was performed immediately in the field using a hand-held blood gas analyzer (epoc®, Siemens Healthcare GmbH, Germany).

### Statistical analysis

Statistical analyses were performed on the R statistical computing software, version 4.0.2 (R Core Team 2020). Reference intervals were calculated following the guidelines published by the ASVCP (23), with modifications based on information from updated modeling studies (25, 26). Histograms for visual inspection of measurand distributions were generated using the R package ggplot2 (27).

Outlier detection, establishment of RIs and calculation of confidence intervals (CI) were undertaken using functions from the referenceInterval package in R (28). Outliers were identified using Horn’s method with Tukey interquartile fences on box-cox transformed dataset (*n* > 30) and Dixon’s Q test method (*n* < =30). If any measurand was identified as an outlier, it was excluded from the set and the statistical analysis was rerun. This procedure was repeated for every measurand being analysed until no more outliers remained. RIs were first calculated for the whole sample dataset collected and subsequently for each sex.

The normality of each measurand was assessed using the Shapiro–Wilk test. A cut-off *p*-value of >0.2 (rather than >0.05) was applied to define a Gaussian distribution for all sample sizes, following recommended guidelines (15, 25). For sample sizes between 40 and 90, if *p* > 0.2, the parametric method was used to generate the 95% reference limits. If *p* ≤ 0.2, the non-parametric method was applied (15, 26). For sample sizes ranging from 20 to 40, reference intervals (RI) were calculated using the non-parametric method, as discrimination between Gaussian and non-Gaussian distributions becomes highly unreliable in this range (26). In such cases, the 2.5 and 97.5% percentiles could not be determined, and the RI limits were directly estimated using the sample minimum and maximum values, i.e., the non-parametric method (29). For sample sizes of 10 to 20, reference values were reported, but RIs were not calculated (23).

The 90% CI of the lower and upper reference limits (RL) were calculated using the bootstrap method. All RIs were established using the singleRefLimit function from the referenceInterval package in R. The ratio of the upper or lower CI to the RI was calculated by dividing the width of the former by the width of the latter.

## Results

**Figure 1:**
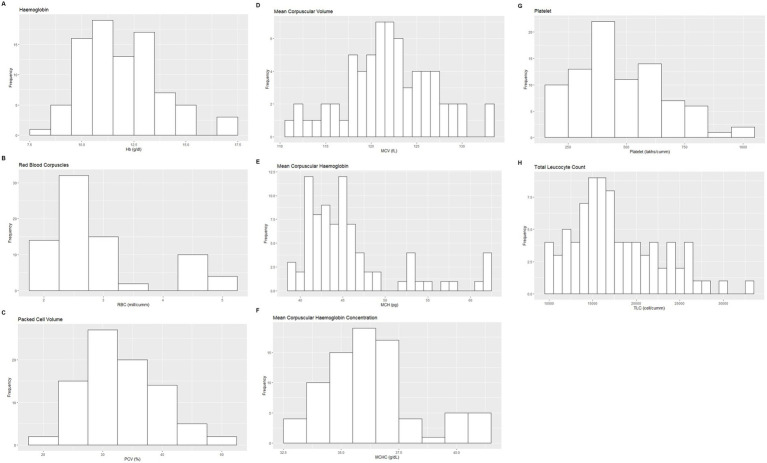
Histograms showing the distribution of results for selected hematology measurands from the Indian elephants (*Elephas maximus indicus*) under human care. The x-axis represents the measurand result; the y-axis represents the frequency of these values occurring.

**Figure 2:**
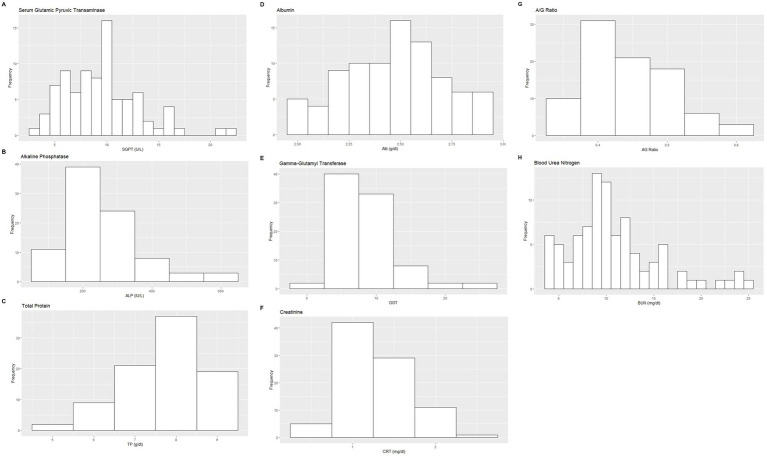
Histograms showing the distribution of results for selected hematology measurands from the Indian elephants (*Elephas maximus indicus*) under human care. The x-axis represents the measurand result; the y-axis represents the frequency of these values occurring.

**Figure 3:**
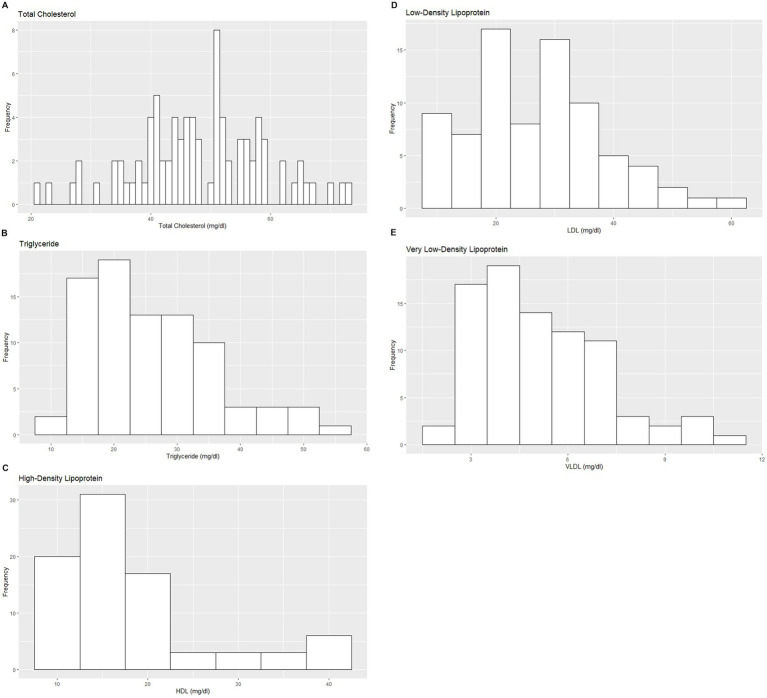
Histograms showing the distribution of results for selected serum lipid profile measurands from the Indian elephants (*Elephas maximus indicus*) under human care. The x-axis represents the measurand result; the y-axis represents the frequency of these values occurring.

**Figure 4:**
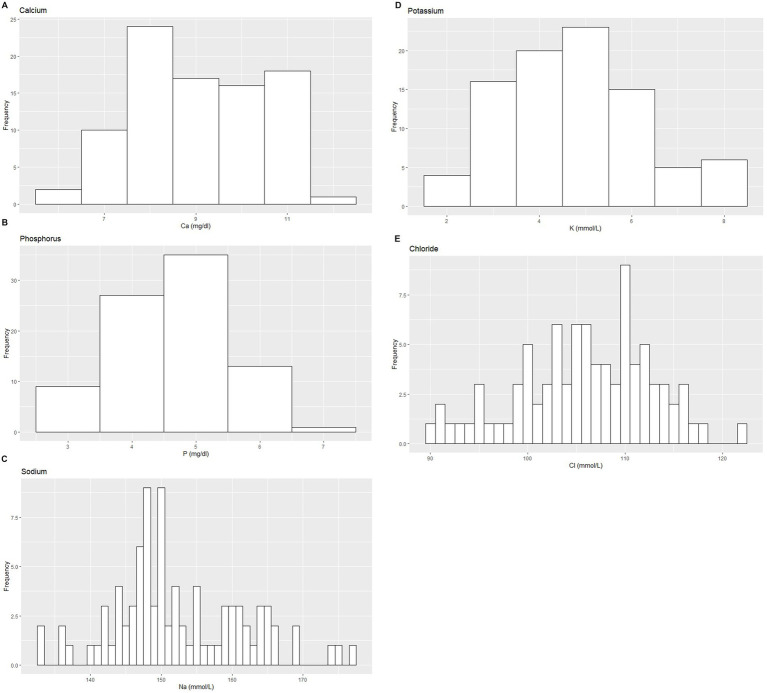
Histograms showing the distribution of results for selected serum electrolyte measurands from the Indian elephants (*Elephas maximus indicus*) under human care. The x-axis represents the measurand result; the y-axis represents the frequency of these values occurring.

Based on the inclusion and exclusion criteria applied in the study, a total of 92 elephants were included in the final analysis. However, the sample size for each analysis varied due to the exclusion of detected outliers across individual measurands and subsets. Histograms for hematology and serum biochemistry measurands are presented in Figures 1–4. Tables 1–7 provide detailed results for hematology, serum biochemistry, and blood gas parameters for the overall population and by sex.

**Table 1:** Hematology reference intervals for Indian elephants (*Elephas maximus indicus*) under Human Care in India.

Measurand (unit)	Sex	*n*	Mean	SD	Median	Min	Max	RI	LRL	URL	*p*-value (*α* = 0.2)	Distribution	Method	♂-♀ *p*-value (*α* = 0.05)
Hb * (g/dl)	All	86	11.91	1.86	11.75	7.8	16.9	8.62–16.78	7.9–9.43	16.66–18.7*	0.07425	NG	NP	0.072
	♂	57	11.52	1.44	11.40	7.8	13.9	8.16–13.9	7.02–8.52*	13.9-14.04	0.1375	NG	NP	
	♀	28	12.51	2.21	12.85	8.7	16.8	8.7–16.8	–	–	–	–	NP	
RBC (×10^12^/L)	All	77	2.91	0.87	2.65	1.8	4.9	1.77–4.9	1.65–1.79	4.9–5.18	1.62E-07	NG	NP	0.276
	♂	46	2.60	0.35	2.60	1.8	3.3	1.92–3.27	1.78–2.06*	3.13-3.418*	0.61	G	P	
	♀	29	3.29	1.38	2.92	1.1	5.6	1.11–5.6	–	–	–	–	NP	
PCV* (%)	All	85	33.03	6.35	31.90	19.0	50	21.73–49.25	18.54–24.46*	48.5-54.5*	0.1253	NG	NP	**0.04**
	♂	57	31.63	4.96	31.40	19.0	41	21.91–41.34	20.06–23.75	39.49–43.19	0.353	G	P	
	♀	29	35.23	8.49	35.50	16.7	50	16.7–50	–	–	–	–	NP	
MCV (fl)	All	67	121.90	4.82	121.70	110.5	133.2	112.49–131.39	110.83–114.14	129.73–133.04	0.9496	G	P	**0.00026**
	♂	41	121.20	3.49	121.20	113.5	128.7	114.39–128.05	112.85–115.92*	126.52-129.59*	0.937	G	P	
	♀	28	129.60	11.80	126.30	114.4	156.4	114.4–156.4	-	-	-	-	NP	
MCH (pg/cell)	All	82	45.94	5.77	44.45	39.1	62.5	39.3–62.39	38.09–39.5	62.27–64.26	1.20E-08	NG	NP	0.315
	♂	50	44.47	3.61	44.00	39.1	54.7	39.15–54.34	38.17–39.21	53.99–55.98	0.0002278	NG	NP	
	♀	28	46.26	5.83	44.90	39.3	62	39.3–62	–	–	–	–	NP	
MCHC (g/dL)	All	80	36.38	1.98	36.10	33.3	41.4	33.40–41	33.2–33.50	40.6–41.40	0.0001729	NG	NP	0.159
	♂	57	36.63	2.04	36.20	33.5	41.4	33.59–41.22	33.18–33.68	41.04–41.64	0.0001624	NG	NP	
	♀	28	35.95	2.57	36.10	32.6	42.7	32.6–42.7	–	–	–	–	NP	
Platelet * (lakhs/cumm)	All	86	477.00	189.97	449.00	150.0	991	171.57–947.1	137.07–193.15	903.2–1074.52*	0.05444	NG	NP	0.069
	♂	58	495.60	173.21	492.50	201.0	991	156.09–835.08	92.16–220.03	771.14–899.01	0.257	G	P	
	♀	29	432.20	217.65	380.00	150.0	966	150–966	–	–	–	–	NP	

^*^The CI (confidence interval of the upper or lower reference limit) to RI ratio exceeded 20%. Bold values in column ‘♂-♀ *p*-value’ indicate a statistically significant difference of the measurand in both sexes.

Hb, hemoglobin concentration; RBC, red blood cell concentration; PCV, packed cell volume; MCV, mean cell volume; MCH, mean cell hemoglobin; MCHC, mean cell hemoglobin concentration; n, number of individuals; SD, standard deviation; Min, minimum; Max, maximum; RI, reference interval; LRL, 90% confidence interval of the lower reference limit; URL, 90% confidence interval of the upper reference limit; *p*-value, probability value; G, Gaussian; NG, non-Gaussian; P, parametric; NP, non-parametric.

**Table 2:** White blood cells and differential leucocyte count reference intervals for Indian elephants (*Elephas maximus indicus*) under human care in India.

Measurand (unit)	Sex	*n*	Mean	SD	Median	Min	Max	RI	LRL	URL	*p-*value (*α* = 0.2)	Distribution	Method	♂-♀ *p*-value (*α* = 0.05)
TLC (cell/cumm)	All	84	17,796	5051.55	16,750	9,900	32,800	9912.5–29,475	9,000–9,925	26,150–32,500*	0.00611	NG	NP	0.462
	♂	57	17,744	5638.96	16,300	9,900	39,800	9,900–35,255	9,000–9,900	30,710–44,410	0.0004072	NG	NP	
	♀	29	18,310	5718.41	16,900	7,700	32,800	7,700–32,800	–	–	–	–	NP	
Neutrophil	All	84	44.78	13.79	41.90	26.6	88.6	27.09–80.71	25.14–27.57	72.82–88.05*	1.42E-05	NG	NP	0.529
	♂	55	44.40	13.22	42.50	19.9	79.4	22.58–78.12	17.36–25.26	76.84–85.4	0.01078	NG	NP	
	♀	29	43.13	13.31	38.50	28.8	80.9	28.8–80.9	–	–	–	–	NP	
Lymphocyte	All	86	35.60	16.40	37.75	5.8	62.4	7.19–60.48	3.03–8.58	58.56–61.83	0.0003317	NG	NP	0.609
	♂	57	35.22	16.29	37.00	5.8	60.5	6.83–60.27	3.94–7.87	60.05–63.82	0.002885	NG	NP	
	♀	29	36.35	16.89	39.00	7.0	62.4	7–62.4	–	–	–	–	NP	
Eosinophil	All	17	10.35	3.18	11.00	3.0	15	–	–	–	–	–	–	
	♂	6	–	–	–	–	–	–	–	–	–	–	–	
	♀	11	10.18	3.46	11.00	3.0	14	–	–	–	–	–	–	
Monocyte	All	70	22.30	18.89	12.15	4.0	79.5	5.24–69.42	2.79–6.48	59.35–80.25*	1.25E-09	NG	NP	0.452
	♂	38	12.01	3.12	11.15	7.9	22.6	7.9–22.6	–	–	–	–	NP	
	♀	20	23.66	19.61	12.65	1.0	54.6	1–54.6	–	–	–	–		
Basophil	All	87	0.00	0.00	0.00	0.0	0	–	–	–	–	–	–	
	♂	58	0.00	0.00	0.00	0.0	0	–	–	–	–	–	–	
	♀	29	0.00	0.00	0.00	0.0	0	–	–	–	–	–	–	

^*^The CI (confidence interval of the upper or lower reference limit) to RI ratio exceeded 20%. Bold values in column ‘♂-♀ *p*-value’ indicate a statistically significant difference of the measurand in both sexes.

TLC, Total Leucocyte Count; n, number of individuals; SD, standard deviation; Min, minimum; Max, maximum; RI, reference interval; LRL, 90% confidence interval of the lower reference limit; URL, 90% confidence interval of the upper reference limit; *p*-value, probability value; G, Gaussian; NG, non-Gaussian; P, parametric; NP, non-parametric.

**Table 3:** Serum biochemistry reference ranges related to muscle and liver health for Indian elephants (*Elephas maximus indicus*) under human care in India.

Measurand (unit)	Sex	*n*	Mean	SD	Median	Min	Max	RI	LRL	URL	*p*-value (*α* = 0.2)	Distribution	Method	♂-♀ *p*-value (*α* = 0.05)
SGPT (U/L)	All	85	9.52	3.65	9.31	3.41	21.60	4.01–20.34	3.45–4.62	19.08–24.78*	0.002987	NG	NP	0.528
	♂	53	8.93	2.42	9.31	4.35	13.70	4.18–13.67	3.24–5.11	12.74–14.61	0.261206	G	P	
	♀	28	9.95	4.36	8.95	4.00	21.00	4.0–21	–	–	–	–	NP	
SGOT (U/L)	All	89	43.67	22.07	41.31	12.10	121.88	14.62–97.01	13.24–17.14	72.14–105.28*	6.88E-05	NG	NP	0.459
	♂	60	45.60	23.59	41.45	15.70	121.88	16.33–109.31	13.59–16.96	96.74–127.11*	1.18E-04	NG	NP	
	♀	29	39.68	18.26	39.20	12.10	82.60	12.1–82.6	–	–	–	–	NP	
ALP (U/L)	All	88	258.10	105.82	234.20	103.20	593.10	124.89–556.68	111.57–146.56	520.25–625.2*	4.36E-06	NG	NP	0.833
	♂	60	256.70	107.25	241.30	132.80	593.10	133.95–574.93	127.81–135.11	556.77–661.26*	2.91E-06	NG	NP	
	♀	28	261.20	104.55	231.60	103.20	499.00	103.23–499	–	–	–	–	NP	
Total Bilirubin (mg/dl)	All	86	0.15	0.05	0.15	0.09	0.38	0.09–0.32	0.088–0.09	0.26–0.39*	4.81E-08	NG	NP	0.511
	♂	57	0.15	0.06	0.14	0.09	0.38	0.09–0.35	0.085–0.09	0.33–0.46*	4.78E-07	NG	NP	
	♀	28	0.15	0.04	0.16	0.09	0.22	0.09–0.22	–	–	–	–	NP	
Direct Bilirubin (mg/dl)	All	88	0.08	0.04	0.07	0.03	0.25	0.04–0.21	0.04–0.05	0.18–0.30*	5.37E-10	NG	NP	0.893
	♂	60	0.08	0.03	0.07	0.03	0.22	0.03–0.21	0.03–0.04	0.20–0.294*	4.42E-07	NG	NP	
	♀	27	0.07	0.03	0.07	0.04	0.13	0.04–0.13	–	–	–	–	NP	
Total Protein (g/dl)	All	88	7.75	0.95	7.99	5.10	9.38	5.54–9.3	4.93–5.98*	9.22-9.53	0.01891	NG	NP	0.112
	♂	59	7.63	0.99	7.90	5.10	9.38	5.3–9.34	4.65–5.5*	9.3-9.73	0.1107	NG	NP	
	♀	29	8.00	0.83	8.10	6.14	9.30	6.14–9.3	–	–	–	–	NP	
Albumin (g/dl)	All	87	2.47	0.24	2.50	2.00	2.92	2–2.91	1.94–2	2.90–2.92	0.09341	NG	NP	**0.0003**
	♂	59	2.41	0.22	2.40	2.00	2.89	1.98–2.84	1.90–2.06	2.76–2.92	0.4109	G	P	
	♀	28	2.61	0.23	2.60	2.13	2.92	2.13–2.92	–	–	–	–	NP	
Globulin (g/dl)	All	87	5.32	0.92	5.40	2.90	6.90	3.36–6.9	2.96–3.82*	6.9-7.12	0.07148	NG	NP	0.836
	♂	58	5.30	0.97	5.45	2.90	6.90	3.09–6.9	2.48–3.28	6.9–7.09	0.1972	NG	NP	
	♀	29	5.37	0.83	5.40	4.00	6.90	4–6.9	–	–	–	–	NP	
GGT (U/L)	All	87	8.12	4.51	7.66	1.91	24.30	2.38–23.18	1.65–2.85	22.06–30.22*	1.96E-06	NG	NP	0.89
	♂	58	7.94	4.30	7.64	2.32	23.70	2.46–22.46	1.9–2.60	21.23–30.09*	8.49E-05	NG	NP	
	♀	28	7.91	3.98	7.60	1.91	19.00	1.91–19	–	–	–	–	NP	

^*^The CI (confidence interval of the upper or lower reference limit) to RI ratio exceeded 20%. Bold values in column ‘♂-♀ p-value’ indicate a statistically significant difference of the measurand in both sexes.

SGPT, serum glutamate pyruvate transaminase; SGOT, serum glutamic-oxaloacetic transaminase; ALP, alkaline phosphatase; GGT, gamma-glutamyltransferase; n, number of individuals; SD, standard deviation; Min, minimum; Max, maximum; RI, reference interval; LRL, 90% confidence interval of the lower reference limit; URL, 90% confidence interval of the upper reference limit; *p*-value, probability value; G, Gaussian; NG, non-Gaussian; P, parametric; NP, non-parametric.

**Table 4:** Serum biochemistry reference ranges related to kidney health for Indian elephants (*Elephas maximus indicus*) under human care in India.

Measurand	Sex	*n*	Mean	SD	Median	Min	Max	RI	LRL	URL	*p*-value (*α* = 0.2)	Distribution	Method	♂-♀ *p*-value (*α* = 0.05)
CRT (mg/dl)	All	88	1.26	0.38	1.23	0.64	2.70	0.65–2.06	0.58–0.66	1.42–2.20*	0.003256	NG	NP	0.225
	♂	59	1.30	0.40	1.26	0.65	2.70	0.66–2.38	0.56–0.67	2.07–2.84	0.02334	NG	NP	
	♀	29	1.19	0.33	1.12	0.64	2.03	0.64–2.03	–	–	–	–	NP	
BUN (mg/dl)	All	89	10.83	4.80	10.00	3.80	24.72	4.12–24.32	3.92–4.45	23.92–27.08	3.68E-05	NG	NP	0.338
	♂	60	10.78	5.26	9.45	3.80	24.72	3.96–24.53	3.61–4.11	24.34–26.62	6.01E-05	NG	NP	
	♀	29	10.94	3.79	10.80	4.31	20.27	4.31–20.27	–	–	–	–	NP	
Albumin/Globulin Ratio	All	89	0.49	0.13	0.50	0.30	0.80	0.3–0.8	0.3–0.3	0.8–0.9	1.34E-05	NG	NP	0.918
	♂	60	0.48	0.13	0.45	0.30	0.80	0.3–0.8	0.3–0.3	0.8–0.85	1.13E-04	NG	NP	
	♀	26	0.47	0.09	0.50	0.30	0.60	0.3–0.6	–	–	–	–	NP	

^*^The CI (confidence interval of the upper or lower reference limit) to RI ratio exceeded 20%. Bold values in column ‘♂-♀ *p*-value’ indicate a statistically significant difference of the measurand in both sexes.

CRT, creatinine; BUN, blood urea nitrogen; n, number of individuals; SD, standard deviation; Min, minimum; Max, maximum; RI, reference interval; LRL, 90% confidence interval of the lower reference limit; URL, 90% confidence interval of the upper reference limit; p-value, probability value; G, Gaussian; NG, non-Gaussian; P, parametric; NP, non-parametric.

**Table 5:** Lipid profile reference ranges for Indian elephants (*Elephas maximus indicus*) under human care in India.

Measurand	Sex	*n*	Mean	SD	Median	Min	Max	RI	LRL	URL	*p*-value (*α* = 0.2)	Distribution	Method	♂-♀ *p*-value (*α* = 0.05)
Total Cholesterol (mg/dl)	All	86	48.17	10.82	47.45	20.70	73.40	26.96–69.39	23.68–30.24	66.11–72.67	0.9184	G	P	0.291
	♂	57	48.99	11.54	47.30	20.70	73.40	26.37–71.61	22.08–30.67	67.31–75.90	0.697751	G	P	
	♀	29	46.57	9.24	47.60	28.30	63.60	28.3–63.6	–	–	–	–	NP	
Triglyceride (mg/dl)	All	84	26.28	10.09	24.60	9.87	56.50	12.55–52.47	11.09–15.23	48.45–61.49*	0.0006691	NG	NP	0.651
	♂	55	25.78	9.62	23.10	9.87	52.50	10.88–51.31	7.97–11.90	50.12–60.02*	0.04407	NG	NP	
	♀	29	27.25	11.04	25.50	13.90	56.50	13.9–56.5	–	–	–	–	NP	
HDL (mg/dl)	All	83	18.40	8.23	16.30	9.00	40.00	9.02–39.38	8.28–9.03	38.76–40.06	9.37E-09	NG	NP	0.46
	♂	56	18.72	8.44	16.80	9.00	40.00	9–39.74	8.49–9	39.49–41.08	4.07E-06	NG	NP	
	♀	27	17.75	7.89	15.70	10.30	39.20	10.3–39.2	–	–	–	–	NP	
LDL (mg/dl)	All	80	26.85	11.34	27.25	7.50	59.50	4.63–49.07	1.06–8.19	45.51–52.64	0.2088	G	P	0.558
	♂	54	27.49	11.52	28.05	7.50	59.50	4.90–50.07	0.49–9.31	45.66–54.48	0.42772	G	P	
	♀	26	25.53	11.05	24.40	8.30	54.60	8.3–54.6	–	–	–	–	NP	
VLDL (mg/dl)	All	84	5.26	2.02	4.90	2.00	11.30	2.52–10.5	2.25–3.05	9.7–12.3*	6.00E-04	NG	NP	0.651
	♂	55	5.16	1.92	4.60	2.00	10.50	2.2–10.26	1.66–2.4	10.02–12.02*	4.15E-02	NG	NP	
	♀	29	5.46	2.21	5.10	2.80	11.30	2.8–11.3	–	–	–	–	NP	
TC/HDL	All	85	2.92	1.01	3.00	1.00	5.30	1.11–5.14	1–1.23	4.98–5.81*	0.1234	NG	NP	0.459
	♂	57	2.98	1.07	3.10	1.00	5.30	0.889–5.075	0.49–1.29	4.68–5.47	0.4446	G	P	
	♀	28	2.78	0.89	2.90	1.20	4.30	1.2–4.3	–	–	–	–	NP	
LDL/HDL	All	84	1.62	0.90	1.70	0.10	3.80	0.1–3.6	0–0.1	3.4–4.12*	0.09903	NG	NP	0.418
	♂	56	1.69	0.95	1.70	0.10	3.80	0–3.55	0–0.19	3.19–3.91*	0.2567	G	P	
	♀	28	1.49	0.79	1.55	0.10	2.90	0.1–2.9	–	–	–	–	NP	

^*^The CI (confidence interval of the upper or lower reference limit) to RI ratio exceeded 20%. Bold values in column ‘♂-♀ *p*-value’ indicate a statistically significant difference of the measurand in both sexes.

HDL, high-density lipoprotein; LDL, low-density lipoprotein; VLDL, very low-density lipoprotein; TC, total cholesterol; n, number of individuals; SD, standard deviation; Min, minimum; Max, maximum; RI, reference interval; LRL, 90% confidence interval of the lower reference limit; URL, 90% confidence interval of the upper reference limit; *p*-value, probability value; G, Gaussian; NG, non-Gaussian; P, parametric; NP, non-parametric.

**Table 6:** Serum electrolyte reference ranges for Indian elephants (*Elephas maximus indicus*) under human care in India.

Measurand	Sex	*n*	Mean	SD	Median	Min	Max	RI	LRL	URL	*p*-value (*α* = 0.2)	Distribution	Method	♂-♀ *p*-value (*α* = 0.05)
Calcium (mg/dl)	All	88	9.04	1.44	9.00	5.60	11.80	6.21–11.38	5.42–6.82*	10.95-11	0.04604	NG	NP	0.163
	♂	60	8.91	1.43	8.70	6.10	11.80	6.36–11.59	5.82–6.62	11.38–11.93	0.07623	NG	NP	
	♀	28	9.31	1.43	9.60	5.60	11.30	5.6–11.3	–	–	–	–	NP	
Phosphorus (mg/dl)	All	85	4.60	0.87	4.62	2.80	6.75	2.89–6.29	2.63–3.16	6.03–6.56	0.6884	G	P	0.527
	♂	58	4.54	0.81	4.61	2.80	6.39	2.957–6.131	2.66–3.26	5.83–6.43	0.866337	G	P	
	♀	27	4.72	0.99	4.64	2.80	6.75	2.8–6.75	–	–	–	–	NP	
Sodium (mmol/L)	All	88	152.40	9.26	150.10	133.10	177.00	133.94–174.77	130.27–134.78	172.55–181.22*	0.05136	NG	NP	0.0503
	♂	59	151.00	9.14	149.80	133.10	177.00	133.25–173	130.1–133.41	169–181	0.1898	NG	NP	
	♀	29	155.30	8.95	154.00	143.70	175.00	143.7–175	–	–	–	–	NP	
Potassium (mmol/L)	All	89	4.82	1.53	4.80	1.80	8.50	1.83–7.81	1.37–2.28	7.36–8.27	0.2364	G	P	0.249
	♂	60	4.69	1.54	4.63	1.80	8.50	1.97–8.5	1.40–2.14	8.5–9.55	0.1784	NG	NP	
	♀	29	5.08	1.49	4.90	2.50	7.70	2.5–7.7	–	–	–	–	NP	
Chloride (mmol/L)	All	89	106.00	6.86	106.40	89.90	121.80	92.56–119.46	90.51–94.60	117.42–121.51	0.3755	G	P	0.251
	♂	60	106.60	6.20	106.80	91.20	117.30	94.451–118.76	92.2–96.70	116.51–121.01	0.276328	G	P	
	♀	29	104.80	8.04	103.60	89.90	121.80	89.9–121.85	–	–	–	–	NP	

^*^The CI (confidence interval of the upper or lower reference limit) to RI ratio exceeded 20%. Bold values in column ‘♂-♀ *p*-value’ indicate a statistically significant difference of the measurand in both sexes.

n, number of individuals; SD, standard deviation; Min, minimum; Max, maximum; RI, reference interval; LRL, 90% confidence interval of the lower reference limit; URL, 90% confidence interval of the upper reference limit; *p*-value, probability value; G, Gaussian; NG, non-Gaussian; P, parametric; NP, non-parametric.

**Table 7:** Blood gas reference ranges for Indian elephants (*Elephas maximus indicus*) under human care in India.

Measurand (unit)	Sex	*n*	Mean	SD	Median	Min	Max	RI	Method
pH	All	33	7.427	0.038	7.422	7.368	7.515	7.368–7.515	NP
pCO_2_ (mmHg)	All	36	39.22	5.042	39.85	28.5	47.9	28.5–47.9	NP
pO_2_ (mmHg)	All	35	92.4	12.983	90.5	71	120.8	71.0–120.8	NP
cHCO_3_ (mmol/L)	All	36	26.32	1.938	25.6	23	30.1	23–30.1	NP
sO_2_ (%)	All	35	97.25	1.268	97.5	94.4	99.6	94.4–99.6	NP
tCO_2_ (mmol/L)	All	36	25.94	1.831	25.3	22.8	29.5	22.8–29.5	NP
BE (ecf) (mmol/L)	All	36	2.136	2.202	1.65	−0.8	8.2	–	NP
BE (b) (mmol/L)	All	36	2.031	2.029	1.75	−0.7	8	–	NP

^*^The CI (confidence interval of the upper or lower reference limit) to RI ratio exceeded 20%.

pCO_2_, partial pressure of carbon dioxide in blood; pO_2_, partial pressure of oxygen in blood; cHCO3, concentration of bicarbonates in arterial blood; sO_2_, oxygen saturation in arterial blood; tCO_2_, total carbon dioxide in arterial blood; BE (ecf), base excess (extracellular fluid); BE (b), base excess (blood); *n*, number of individuals; SD, standard deviation; Min, minimum; Max, maximum; RI, reference interval; NP, non-parametric.

For all statistically analyzed measurands, the sample size (n), mean, standard deviation (SD), median, minimum (Min), and maximum (Max) values are reported. RI values are accompanied by the 90% confidence intervals for the lower and upper reference limits (LRL and URL), where applicable. Additionally, the frequency distribution type for each measurand, either Gaussian (G) or Non-Gaussian (NG) is indicated, along with the statistical method used for calculations, whether parametric (P) or non-parametric (NP).

The present study employed an automated veterinary hematology analyzer to establish reference RIs for Differential Leucocyte Count (DLC) in elephants. However, manual DLC methods are recognized as more accurate and reliable for this species. Due to technical constraints, manual DLC evaluation was not performed in this investigation. Consequently, the DLC results presented here should be interpreted with caution, and future studies should prioritize manual validation for greater precision.

## Discussion

This study establishes RIs for hematology, serum biochemistry, and blood gas parameters in Indian elephants under human care, with the aim of providing essential baseline data for veterinarians and wildlife managers working with this species. Although the reference RIs presented are derived from elephants in a managed setting, the husbandry practices for these animals were semi-captive in nature. Each elephant had access to natural forest habitats for at least 12 h daily, allowing them to engage in natural behavior and forage without human intervention during this time. This setup provided a more naturalistic environment, making these RIs relevant for assessing the health status of free-ranging Indian elephants in the region to a certain degree.

The study adopted a two-pronged approach to develop RIs that accurately represent a truly healthy Indian elephant population (23). The first approach involved excluding elephants with physical ailments, based on thorough physical examinations and clinical histories, as well as those experiencing physiological variations such as work-related stress, musth, pregnancy, or lactation. Given that chemical immobilization and the associated capture stress can alter various blood parameters and potentially impact the accuracy of RIs, all animals in this study were sampled without the use of any form of sedation (15, 30, 31). Additionally, uniform sampling protocols and laboratory procedures were implemented to minimize variability (23). In the second stage, statistical analysis was applied to rigorously eliminate outliers within each measured parameter, ensuring the reliability of the derived RIs for the species (15, 23, 25, 26).

To the best of our knowledge, this study is the first of its kind on the subspecies from the Indian subcontinent and the second globally for the species to generate RIs in accordance with ASVCP guidelines. The only other study adhering to these guidelines was conducted on semi-captive working Asian elephants in Myanmar (22). While numerous studies have reported hematology and biochemistry ranges for Asian elephants (16–20, 32, 33), several limitations restrict their utility for establishing reliable RIs for the subspecies. Among these studies, only those by Dos Santos and co-workers (22) and Janyamethakul et al. (21) involved sample sizes exceeding 100 individuals. Most other studies had sample sizes ranging from as few as six elephants (32) to a maximum of 50 elephants (20). Although Dos Santos et al. (22) sampled 765 elephants, it is noteworthy that most of the individuals were sampled multiple times.

In Indian studies, the protocols for sampling, storage, transportation, and analytical methods are either not described or exhibit considerable variability across studies. Most of these studies report hematology reference values and some clinical chemistry parameters as mean or median values with standard deviations or ranges. However, they lack rigorous statistical analyses, such as outlier identification, exclusion, and the calculation of RIs or confidence intervals (CIs) for the reported limits (16–20, 32, 33). A similar trend is evident in available RI studies for African elephants. Although numerous hematology and biochemistry studies have been conducted on African elephant populations, only the recent study on free-ranging African elephants from Kruger National Park, South Africa, appears to have generated RIs in accordance with the ASVCP guidelines (15).

Hematology parameters, such as red blood cell (RBC) count, packed cell volume (PCV), and hemoglobin (Hb) levels, are often higher in males due to testosterone’s stimulatory effect on erythropoiesis (22, 34–36). However, in this study, RBC count and Hb levels were similar between sexes, while PCV showed significant variation (males: 21.91–41.34%; females: 16.7–50%; *p* = 0.04). As testosterone levels were not measured, the potential effects of androgens on erythropoiesis remain unexplored, warranting further investigation. Although hematology values are known to vary by geographical region (15, 22), comparisons with available data from Myanmar elephants showed that PCV (RI: 29–42%) and hemoglobin (RI: 8.5–17.0 g/dL) align closely with the findings of the current study (22). Similarly, a recent elephant hematology study from India, which did not include outlier removal, reported minimum-maximum ranges for PCV (28.3–54%), hemoglobin (Hb: 10.1–17.8 g/dL), RBC (2.1–3.74 × 10^6^/μL), and RBC indices (MCV: 90.90–161.90 fL; MCH: 30.33–56.74 pg.; MCHC: 20.00–46.64 g/dL) that were largely comparable to the findings of this study (19). However, these reported ranges are much wider compared to the RIs established in the current study, underscoring the critical importance of identifying and excluding outliers and employing robust statistical analyses, as recommended by ASVCP guidelines, in the establishment of reliable RIs.

The WBC counts in Asian elephants show significant variability across studies, with values ranging from a minimum of 7.2 × 10^9^/L in females (21) to a maximum of 29.7 × 10^9^/L in working elephants from Myanmar (22). In the present study, the WBC RIs generated (9.9–29.5 × 10^9^/L) align well with the WBC RIs reported for Myanmar elephants (7.7–29.7 × 10^9^/L) (22), though they are notably higher than those reported for African elephants (6.6–15.7 × 10^9^/L) (15). Other studies on working Asian elephants report WBC RIs of 7.92–21.90 × 10^9^/L in males and 7.20–23.22 × 10^9^/L in females from Thailand (21) and 7.6–20.8 × 10^9^/L in southern India (19). However, both these studies lacked advanced statistical analysis. Notably, despite most previous studies employing manual methods to assess WBC levels, the present study utilized an automated veterinary hematology analyzer and generated RIs consistent with other Asian elephant studies, including the Myanmar study conducted in accordance with ASVCP guidelines. While these findings underscore the potential of automated hematology analyzers for rapid WBC evaluation in elephants, further validation through detailed investigations is necessary to establish their reliability and accuracy (15, 37, 38).

Similar to WBC counts, the present study utilized an automated veterinary hematology analyzer to establish RIs for DLC. Notably, elephants, as members of the Mirorder *Tethytheria*, exhibit significant morphological variations in their leukocytes compared to other mammalian clades (15, 39, 40). These variations include the presence of bilobed and round monocytes (15, 41) and heterophils instead of neutrophils, a characteristic shared with other extant species within the Grandorder *Paenungulata* (40, 42, 43). Automated hematology analyzers may misclassify such unique leukocyte morphologies, potentially leading to inaccurate or biased DLC results (37, 38, 41, 44, 45). Due to technical limitations, the manual method for DLC evaluation was not performed in this study, precluding a direct comparison between the current findings and those of previous Asian elephant hematology studies. We acknowledge this as a limitation of the current study and concur with previous research indicating that manual DLC methods are more accurate and reliable for elephants, provided there is adequate expertise in identifying and classifying the various leukocyte types (15, 41, 45).

The platelet RIs established in this study (171.57–947.1 × 10^9^/L) were notably higher than those reported in a study from Thailand (101–590 × 10^9^/L) (21) and exceeded the highest previously reported count of 719 × 10^9^/L (41). The observed platelet count variations may have been influenced by multiple factors, including platelet clumping (15, 41) and methodological differences in analytical approaches between studies (45). Platelet estimation in elephants is challenging, as their small, highly reactive platelets demonstrate increased aggregation potential, which may lead to artificially depressed automated counts (45). Additional confounding factors include platelet activation during phlebotomy and potential EDTA-dependent pseudothrombocytopenia. While the current study obtained platelet counts at the higher end of the reported range, suggesting minimal clumping effects in samples, it should be noted that methodological differences may have accounted for some variation between studies use for comparison. The clinical significance of accurate platelet quantification thus underscores the importance of manual validation for both leukocyte differentials and platelet estimates in elephant hematology (45).

The clinical chemistry panels used to assess muscle and liver health in elephants include creatine kinase (CK), SGOT, SGPT, ALP, and GGT (21, 46). Although CK levels were not evaluated in this study, SGOT and ALP levels were notably higher than the reported ranges for the species (21, 22, 46). SGOT, which is widely distributed in skeletal and cardiac muscles, the liver, and erythrocytes, typically increases due to hepatocellular injuries (46). While the effects of physical activity on SGOT in elephants are not well understood and are primarily extrapolated from human or companion animal studies (47), recent research indicates that SGOT and ALP levels tend to rise over time with a decrease in physical activity (46). In contrast, the elevated SGOT and ALP levels observed in this study were attributed to higher values in certain Kumki males (121.88 U/L AST and 593.10 U/L ALP), which exhibited greater physical activity compared to the rest of the study animals, potentially indicating underlying muscular damage.

The SGPT is generally considered less diagnostically significant in elephants due to its low serum levels (48). However, the SGPT levels in this study (4.01–20.34 U/L) were slightly higher compared to available data for Asian elephants. GGT, a biliary enzyme, is monitored to assess hepatobiliary health. The RI for GGT in this study (2.38–23.18 U/L) fell within the range reported for Indian elephants (33, 48) but was slightly higher than values for African elephants (15). Notably, serum ALP and GGT activities are significantly elevated in Asian elephant males during musth periods (22). However, none of the parameters in this study showed significant differences between sexes, likely due to the exclusion criteria that ensured no elephants with physiological variations, such as musth, were included.

The serum protein components, such as TP, albumin, and globulin fractions, are useful for evaluating inflammatory responses in elephants (49). The TP and albumin values in this study were consistent with reported ranges for Asian elephants, both in managed and free ranging conditions (22, 49, 50), though slightly lower than those for African elephants (15). While geographical, seasonal, and forage related factors are known to influence serum protein levels, the observed levels in Asian elephants across various studies, including non-native zoological settings, remain remarkably consistent (15, 22, 49, 50). Therefore, any significant deviation from the current RI values (5.54–9.3 g/dL) may indicate an underlying pathology.

Although serum clinical chemistry panals for assessing kidney health are not fully understood in elephants, and urine analysis is often considered a more reliable method, parameters such as BUN, CRT, TP, and albumin-to-globulin ratios can still serve as early indicators of changes in hydration status and renal efficiency (51, 52). Additionally, since the kidneys play a critical role in regulating blood electrolytes, assays for NA, K, and Cl can provide valuable clinical insights when interpreted alongside renal-specific biochemistry panels (15, 53). In the current study, the observed serum CRT and BUN levels ranged from 0.65–2.06 mg/dL and 4.12–24.32 mg/dL, respectively, which align with values reported for Asian elephants under human care (21, 51). Similarly, serum electrolytes such as calcium (6.21–11.38 mg/dL), sodium (133.94–174.77 mmol/L), potassium (1.83–7.81 mmol/L), and chloride (92.56–119.46 mmol/L) were within the ranges documented for managed Asian elephants (51). However, serum phosphorus levels could not be compared due to a lack of data from other studies on Asian elephants. Additionally, magnesium levels were not included in this study because the test was not available in the predefined profile of the automated biochemistry analyzer used.

Serum electrolyte values are also a valuable tool for assessing nutritional status and preventing mineral deficiencies, which can have significant downstream effects on metabolic and developmental processes in managed settings. Studies on African elephants have reported considerable variability in serum electrolyte concentrations across different populations and research efforts (15, 54, 55). These variations are likely influenced not only by clinical factors but also by seasonal, geographical, and nutritional variations, all of which are known to impact serum electrolyte levels (15). Such variability underscores the importance of context-specific interpretations and highlights the need for further research to establish robust, population-specific RIs for elephants across diverse habitats and management conditions. Additionally, caution should be exercised when applying reference intervals to free-ranging elephants, as their dietary intake can fluctuate significantly with seasonal and environmental changes.

Unlike other simple-stomached species, lipid profiles in Asian elephants remain poorly utilized for diagnostic purposes. Studies on lipid profile RIs are also limited and lack comparability with the present study, as the only available research focuses exclusively on female Asian elephants used for tourism in Thailand (56). Nevertheless, the same study identified significant associations between adrenal and metabolic hormones and lipid profiles, highlighting their potential utility in assessing health and welfare in managed elephant populations.

The current study is the first to establish baseline arterial blood gas (ABG) controls for Asian elephants in their native range. ABG values in elephants are known to be influenced by body position due to the species’ unique anatomy, with values varying significantly between upright and recumbent positions (57). In India, free-ranging elephants, particularly managed populations and wild females, are often immobilized using standing sedation with α2 adrenergic agonists (58). However, the capture of free-ranging males typically involves potent narcotics, resulting in sternal recumbency. Given the substantial body mass and the potential side effects of opioids, recumbency during immobilization can adversely affect cardiopulmonary functions in elephants (59). Thus, establishing baseline ABG parameters in conscious, non-sedated elephants is essential for identifying physiological abnormalities during field capture procedures. Although ABG values in upright positions were not evaluated as all samples were collected under lateral recumbency in the current study, the results align closely with those from a study on female Asian elephants in managed settings in the USA (49). The observed pO2 (71.0–120.8 mmHg), pCO2 (28.5–47.9 mmHg), and sO2 (94.4–99.6%) values indicate that elephants in lateral recumbency do exhibit some degree of hypoxia and hypercapnia even without sedation. These findings underscore the importance of actively supplementing oxygen during captures, particularly when opioids are used without tranquilizers, as is common practice in India (58).

The study has limitations with respect to the skewed representation of female elephants in the sample population. The cohort predominantly comprised males, as they are preferred for patrolling and Kumki work, resulting in a smaller sample size for females. For most parameters, the female sample size ranged from 20 to 40, necessitating the use of non-parametric methods to calculate RIs. Consequently, the RIs for most parameters lack lower (LRLs) and upper reference limits (URLs). Additionally, larger sample sizes would have enabled partitioning by age classes, which was not feasible in this study. Future studies incorporating these aspects are warranted. Nevertheless, we propose that the established RIs remain clinically significant and can serve as valuable tools for assessing the health, nutritional status, and husbandry management of Asian elephants in the region, including zoological settings. The study population, maintained in near-natural conditions with free-choice fodder and activities mimicking the natural behavior of free-ranging elephants, enhances the relevance of these RIs for free-ranging populations as well.

## Conclusion

This study establishes the first comprehensive RIs for hematology, serum biochemistry, and blood gas parameters in Indian elephants under semi-captive conditions, providing critical baseline data for health assessments in both managed and free-ranging populations in the region. By employing rigorous exclusion criteria, uniform sampling protocols, and advanced statistical methods, the derived RIs offer a reliable framework for evaluating the physiological and nutritional status of elephants, particularly in areas with limited diagnostic infrastructure. The findings highlight the importance of context-specific RIs, as variations in hematology and biochemistry parameters are influenced by various intrinsic and extrinsic factors. While the skewed representation of females, lack of age-class partitioning, and absence of manual DLC counts remain limitations, the established RIs are clinically significant and applicable to both zoological and free-ranging settings in the region. Future studies should focus on expanding sample sizes, incorporating age and sex-specific analyses, and validating automated diagnostic tools to further refine these RIs and enhance their utility in elephant conservation and management.

## Data Availability

The original contributions presented in the study are included in the article/supplementary material, further inquiries can be directed to the corresponding authors.
